# Hydrogels for Cartilage Regeneration, from Polysaccharides to Hybrids

**DOI:** 10.3390/polym9120671

**Published:** 2017-12-04

**Authors:** Daniela Anahí Sánchez-Téllez, Lucía Téllez-Jurado, Luís María Rodríguez-Lorenzo

**Affiliations:** 1 Instituto Politécnico Nacional-ESIQIE, Depto. Ing. en Metalurgia y Materiales, UPALM-Zacatenco, Mexico City 07738, Mexico; ltellezj@ipn.mx; 2 Networking Biomedical Research Centre in Bioengineering, Biomaterials and Nanomedicine, Centro de Investigación Biomédica en Red—Bioingeniería, Biomateriales y Nanomedicina (CIBER-BBN), Av. Monforte de Lemos 3-5, Pabellón 11, Planta 0, 28029 Madrid, Spain; luis.rodriguez-lorenzo@ictp.csic.es; 3 Department Polymeric Nanomaterials and Biomaterials, ICTP-CSIC, Juan de la Cierva 3, 28006 Madrid, Spain

**Keywords:** cartilage regeneration, polymeric hydrogels, polysaccharides, hybrid hydrogels, hybrid scaffolds

## Abstract

The aims of this paper are: (1) to review the current state of the art in the field of cartilage substitution and regeneration; (2) to examine the patented biomaterials being used in preclinical and clinical stages; (3) to explore the potential of polymeric hydrogels for these applications and the reasons that hinder their clinical success. The studies about hydrogels used as potential biomaterials selected for this review are divided into the two major trends in tissue engineering: (1) the use of cell-free biomaterials; and (2) the use of cell seeded biomaterials. Preparation techniques and resulting hydrogel properties are also reviewed. More recent proposals, based on the combination of different polymers and the hybridization process to improve the properties of these materials, are also reviewed. The combination of elements such as scaffolds (cellular solids), matrices (hydrogel-based), growth factors and mechanical stimuli is needed to optimize properties of the required materials in order to facilitate tissue formation, cartilage regeneration and final clinical application. Polymer combinations and hybrids are the most promising materials for this application. Hybrid scaffolds may maximize cell growth and local tissue integration by forming cartilage-like tissue with biomimetic features.

## 1. Introduction: Current Clinical Approaches and the Need for New Developments

The aim of this paper is to review the current state of the art of materials for cartilage substitution and regeneration. Section 1 describes the current state of the art in clinical treatments. Polymeric hydrogels used in cartilage regeneration and the reasons hindering their clinical success are reviewed in Section 2. The preparation techniques using polysaccharides and the resulting hydrogel properties are described in Section 3. Finally, future trends are explored in Section 4.

Several reviews about hydrogels for cartilage regeneration have been published for the last 10 years, focusing on preparation and characterization, natural and synthetic polymer precursors, gelation kinetics, cell and drug delivery, growth factors, mechanical properties and biocompatibility. Nevertheless, most of those reviews do not propose new alternatives to improve hydrogels properties which can fulfill the real clinical needs in terms of tissue regeneration, mechanical properties and degradation kinetics. Therefore, this paper reviews relevant literature published during 2013–2017, related to the application, fabrication, characterization, in vitro and in vivo assays of biomaterials based on hydrogels for cartilage regeneration. The studies selected are articles and reviews written in English.

**Figure 1:**
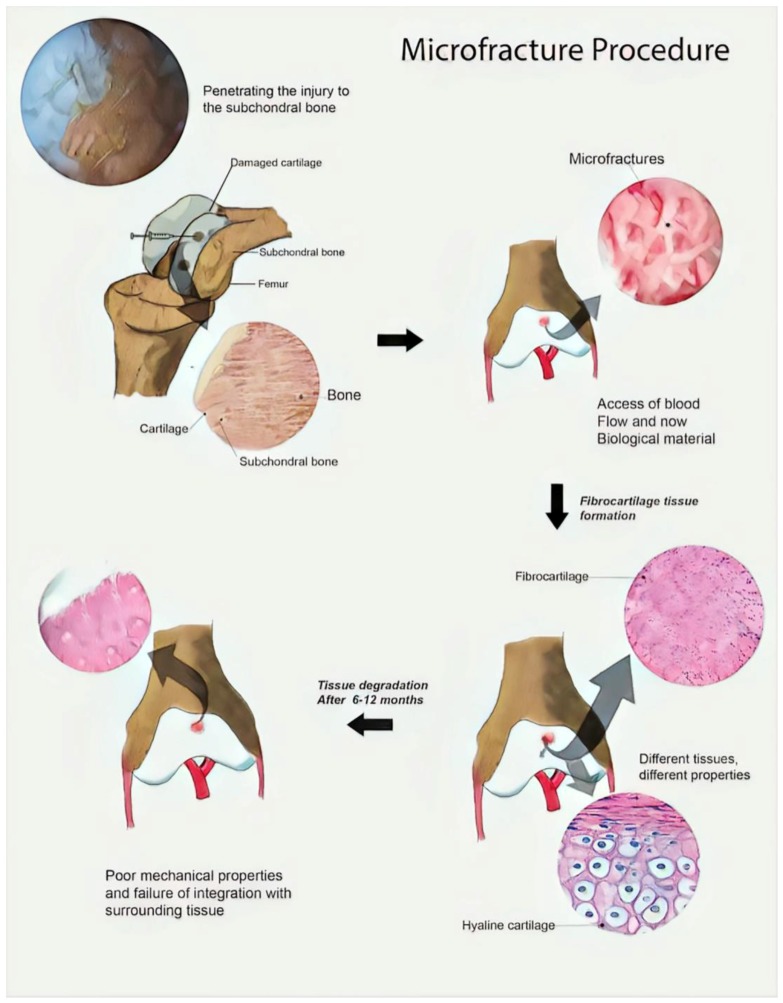
Common clinical procedure used to heal cartilage injury. Original illustration designed and provided by the authors.

Currently, there are no clinical satisfactory solutions for cartilage tissue regeneration [1,2,3]. The most widely used clinical procedure to heal cartilage injury involves penetrating the wound to the subchondral bone, allowing the access of blood flow and new biological material [4,5,6,7]. However, such clinical treatments often result in the formation of fibrocartilaginous tissue (Figure 1), which is weaker than the original one, failing to integrate properly with surrounding tissue, and degrading over a period between 6 to 12 months [8,9,10,11,12].

**Figure 2:**
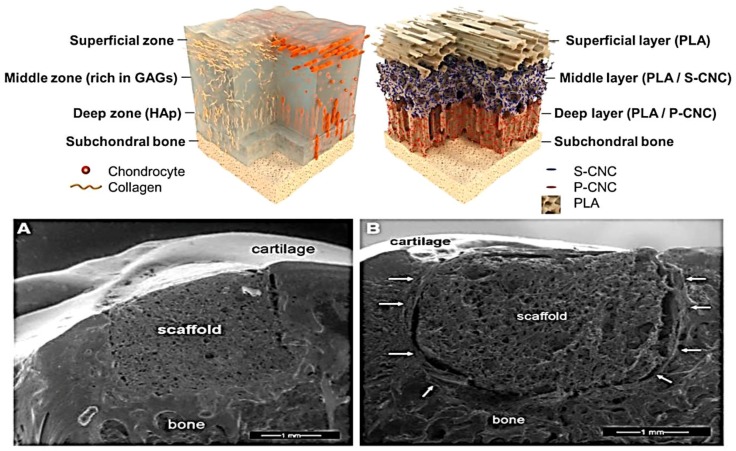
**Top**. How artificial scaffolds try to mimic the anisotropic characteristic in cartilage tissue [33,34]. Reprinted with permission of [33] Sandra Camarero-Espinosa et al. *Biomaterials*, 74: 42–52. **Bottom**. Matrix regeneration within a macroporous non-degradable implant for osteochondral defects is not enhanced with partial enzymatic digestion of the surrounding tissue. (**A**) Environmental scanning electron microscopy of a longitudinal slice taken through the cartilage-bone-implant. The specimen was retrieved at the 1 month post-operative time point. Good integration between the implant and the surrounding bone and articular cartilage was observed. (**B**) Environmental scanning electron microscopy image of a longitudinal slice taken through the cartilage-bone-implant construct at 3 months. Fibrous encapsulation of the implant is highlighted by arrows. Reprinted with permission of [34] Aaron J. Krych et al. *J. Mater. Sci. Mater. Med.*, 24: 2429–2437.

During the last few years, material scientists and tissue engineers have tried to help clinicians by confronting the challenge of manufacturing porous 3D scaffolds which resemble the chemical composition and architecture (Figure 2) of the extracellular matrix (ECM) of the cartilage [13,14,15,16,17,18,19]. Most of the studies are directed to determine how chemical composition and architecture influence cellular phenotype, differentiation, integration and extracellular matrix secretion during in vitro [20,21,22,23,24,25] and in vivo [26,27,28,29,30,31,32] assays.

Several natural and synthetic polymers are being used to create novel materials, as collected in Table 1, in attempts to produce scaffolds for tissue engineering and regenerative medicine having clinical application [35].

However, the complexity of the physical structure and properties of cartilage, including mechanical [50,51,52,53,54], anisotropic [55,56,57], nonlinear [58,59,60], inhomogeneous [61,62,63] and viscoelastic properties [64,65,66], are thought to be directly related to the failing of most of the attempts made to fabricate artificial substitutes for cartilage [67,68,69,70,71]. As a consequence, there are not yet biomaterials for cartilage regeneration in clinical use with satisfactory results. Scaffolds based on tissue-engineered constructs, osteochondral biomimetic scaffolds, cell-free biphasic or three-phasic scaffolds, autologous scaffolds, engineered-tissue grafts, porous implants have not demonstrated to be a satisfactory solution in clinical application. Therefore, it seems evident that there is a need to designi more suitable scaffolds and to develop new types of materials which can be used for cartilage regeneration.

In the case of articular cartilage repair, the required materials must provide successful mechanical properties, biological delivery, fixation of the device in situ and stability to the joint. Besides, the assays done to these materials need to be based on the intended biological effect and potential risks which have to be evaluated, such as toxicity, dedifferentiation, immunogenicity and contamination. Some preclinical trials with little animal models, such as rats and white New Zealand rabbits, are necessary to predict how biomaterials may behave during clinical assays. Moreover, some preliminary studies can be done in cadaveric human bodies; however extensive trials with large animal preclinical models are mandatory to obtain market approval. Therefore, just a few of the novel materials or tissue protocols are allowed to have clinical application or even to be commercialized. When trying to compare different studies of novel materials once they are introduced into clinical practice, there are other problems: (1) lack of homogeneity due to the different studied population; (2) short- and mid-follow-ups; (3) use of different evaluation systems; (4) new scaffold-based strategies for cartilage regeneration, either cell seeded or cell-free biomaterials; (5) procedures which differ in scaffold fixation methods, surgical approaches and postoperative rehabilitation phases. Therefore, there is the need among scientists, clinicians, industry and regulatory experts to improve communication and collaboration in order to overcome all the barriers in tissue engineering and to establish a defined road map to reach clinical application.

## 2. Hydrogels in Cartilage Regeneration

**Figure 3:**
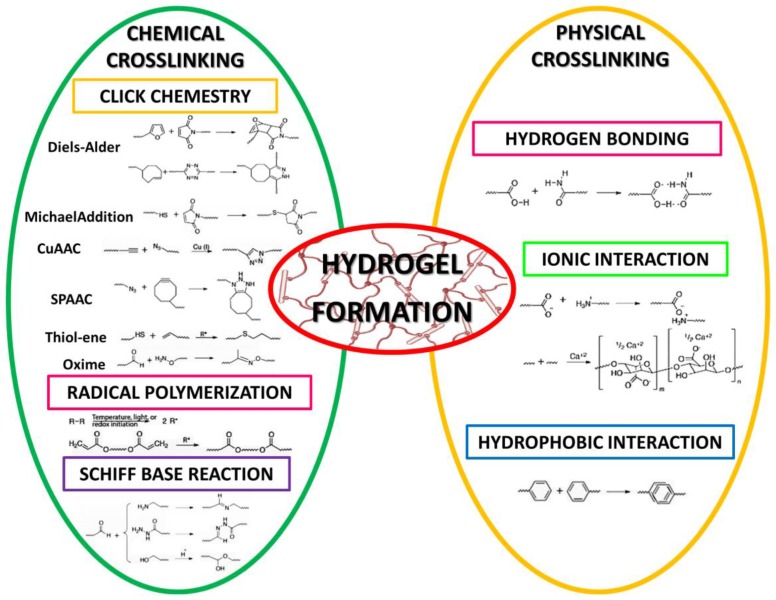
Chemical and physical crosslinking methods used to form hydrogels.

Hydrogels are emergent candidates for applications in cartilage regeneration. Hydrogels are three-dimensional hydrophilic polymer networks made up of water-soluble polymers, crosslinked by either covalent or physical methods [72] (Figure 3) to form a water-insoluble hydrogel [73,74]. Hydrogels can be composed of natural polysaccharides [75,76,77], proteins [78,79,80,81,82] or synthetic polymers [83,84,85]. Hydrogels are able to swell and retain great portions of water, from 20% to 99% by weight, when placed in aqueous solutions [86,87,88]. Hydrogels, tested as matrices to build up scaffolds, provide highly desirable 3D environments for cell growth, holding a great promise for the regeneration of cartilaginous tissue as in vitro and in vivo studies showed [89,90,91,92,93,94]. Several studies use cells to catalyze tissue formation while being distributed in 3D hydrogel matrices. Cell matrix adhesion to hydrogel is an important interaction which regulates stem cell survival, self-renewal, and differentiation. Using 3D culture systems (hydrogels) may provide an appropriate niche, scaffolding and environmental bioactive signals for cells. Depending on their physical structure and chemical composition, hydrogels can preserve a compositional and mechanical similarity with the native extracellular matrix of cartilage [95,96,97,98]. These properties are necessary for controlling cell response, differentiation and functional tissue regeneration [99,100,101,102,103,104,105,106].

One important reason for the choice of hydrogels in cartilage applications is the possibility of making them injectable which offers advantages over solid scaffolds such as the possibility of using a non-invasive approach. Injectable hydrogels can fill any shape defect and they may provide a homogeneous cell distribution within any defect size or shape prior to gelation [107,108,109,110,111]. Over recent years, a variety of naturally [112,113,114,115,116,117,118,119] and synthetically [120,121,122] derived materials such as silk [123,124], resilin [125], chitosan [126], chondroitin sulfate [127], hyaluronic acid [128,129,130,131], gelatin [132], agarose [133], alginate [134] poly(vinyl alcohol) (PVA) [95,135] poly(acrylic acid) [136], acrylamide [137] and many others have been used to form injectable hydrogels for cartilage repair.

Collagen II and glycosaminoglycans (GAGs) are cartilage-specific extracellular matrix components; they play a crucial role in regulating the expression of chondrocytic phenotype and in supporting chondrogenesis. They have been used for in vitro and in vivo assays [138]; many attempts have been made with different GAGs precursors of hydrogels to provide an appropriate biochemical and biomechanical environment for cells [139,140,141,142,143,144,145,146]. Unfortunately, hydrogels derived from GAGs degrade really fast, thus different chemical modifications have to be introduced. Several crosslinking degrees are necessary in attempt to modulate their degradation kinetics. However, their biological response is also modified [147,148,149,150,151]. In general, the studies found on literature can be divided into two main types: (1) cell-free hydrogel scaffolds; and (2) cell-seeded hydrogel scaffolds.

### 2.1. Cell-Free Hydrogel Scaffolds

Investigations based on cell-free hydrogel scaffolds focus on their physico-chemical characterization and mechanical properties [152]. These studies allow a full understanding of physical and chemical interactions within materials and how these interactions may affect biological and mechanical properties of scaffolds.

Poly(ethylene glycol) (PEG) and derived hydrogels are ones of the most widely used synthetic polymer for tissue engineering. The modulus for bovine articular cartilage, measured in compression mode, is 950 KPa, which is close to the value of the fully hydrated polyethylene glycol diacrylate (PEG-DA) hydrogel [87]. Polyvinyl alcohol is another synthetic polymer widely used to form hydrogels due to their excellent biocompatibility, high permeability to fluids (showing an equilibrium water content of 32 ± 5%) and low friction coefficients (µ) in the range of 0.02 to 0.05 against smooth and wet substances. Some studies on PVA-based scaffolds aim to demonstrate that under tribological loading, friction and wear characteristics compatible to natural articular cartilage can be achieved [153]. As low friction coefficients are required for engineered cartilage, polyvinyl alcohol (PVA)/polyvinylpyrrolidone (PVP) hydrogels were synthesized with different polymerization degrees: 1700, 2400 and 2600 for the PVA; and different polymer concentrations: 10% *w*/*w*, 15% *w*/*w* and 20% *w*/*w* of PVA/PVP. It was found that the inner structures of hydrogels tend to be denser when polymer concentration and polymerization degree of PVA increase. While the friction coefficient increases (from 0.037 to 0.044) with an increment in the polymerization degree of PVA (average increase rate is approximate 3%), the friction coefficient decreases (from 0.033 to 0.03 for a 2.5 N load; from 0.049 to 0.045 for a 7.5 N load) with an increment in the polymer concentration (from 10% to 20%) in the low load region and under liquid lubrication. Thus, there is the need to keep friction coefficients stable under lubricated conditions [154].

Another study using PVA-based hydrogels, crosslinked with trimetaphosphate (STMP), revealed that fully hydrated hydrogels were covalently crosslinked systems when mechanically tested, with a rheological behavior (the *G*′ changed from 0.01 MPa (0.01 Hz) to 0.02 MPa (15 Hz)) similar to that of tibia cartilage (*G*′ = 0.03 for tissue surface and *G*′ = 0.11 for overall tissue) [86]. As previously said, it is an important challenge to develop scaffolds which possess mechanical properties mimicking those of cartilage tissue, since cartilage is a complex nonlinear, viscoelastic and anisotropic extracellular matrix structure. T. Chen et al. [155] reported that hydrodynamic conditions, simulating the motion-induced flow fields between the articular surfaces within the synovial joint, induce the formation of a distinct superficial layer on tissue engineered cartilage hydrogels. These hydrodynamic conditions enhance, on the superficial layers, the production of cartilage matrix proteoglycan, type II collagen and a highly aligned fibrillary matrix which resembles the alignment pattern in native tissue surface zone.

Since many materials do not exhibit a low friction coefficient or withstand several loading cycles, some of them are infiltrated with an interpenetrating network hydrogel to form functional scaffolds which provide load-bearing and tribological properties, similar to native cartilage ones. For example: (1) a porous three-dimensionally woven poly(ε-caprolactone) fiber scaffold was infused with a “tough-gel” made of alginate and polyacrylamide [83]; (2) a boundary lubricant functionalized PVA-based hydrogel was developed to be used as a synthetic replacement for focal defects in articular cartilage [156]. Other techniques to develop hydrogels with high mechanical strength are: by using a double network or two-step polymerization [88,157]; or by functionalizing hydrogels with different organic or inorganic molecules [158].

### 2.2. Cell-Seeded Hydrogel Scaffolds

**Figure 4:**
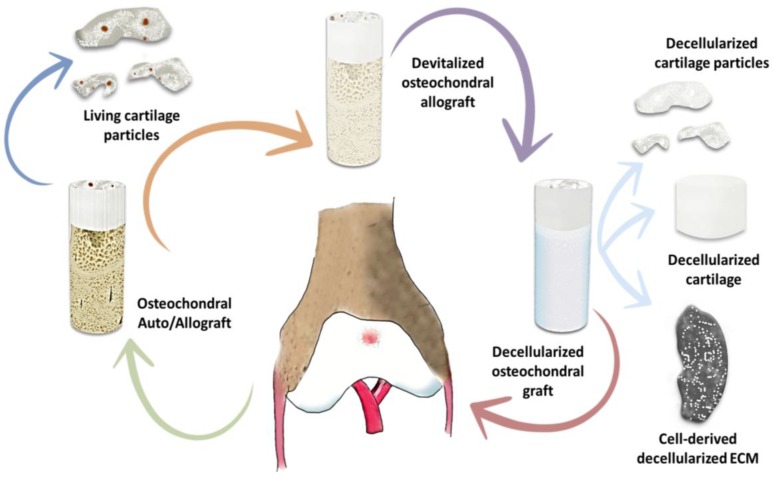
Matrix-based scaffold approaches for cartilage regeneration. Original illustration designed and provided by the authors.

Cell-based hydrogel scaffold therapy is one of the main strategies being investigated in cartilage regeneration. Several scaffolds and materials are being evaluated. These studies focus on whether or not the hydrogels provide an appropriate biochemical and biomechanical environment for a long-lasting hyaline-type cartilage regeneration. Decellularized extracellular matrices from natural tissues like dermis or adipose are being studied as functional biologic scaffolds (Figure 4). It is possible to ensure the bioactivity of a substrate when scaffolds are seeded with a specific cell type, either chondrocytes or stem cells, and if these cells are able to proliferate regardless of “natural” conditions. As an example, Giavaresi G. et al. [159] evaluated in vitro the biological influence of a decellularized human dermal extracellular matrix on human chondrocytes (NHAC-kn) and mesenchymal stromal cells (hMSC). The study showed that at 24 h after seeding, cells adhered consistently to dermal membranes (NHAC-kn = 93% and hMSC = 98%); at 7 days, cell viability index was 98% for both cell cultures seeded on dermal membranes; and after 14 days of culture, the indexes increased significantly for both cell cultures (*p* < 0.0005; NHAC-kn = 136% and hMSC = 263%). Furthermore, a biohybrid composite scaffold, composed by combining a decellularized Wharton’s jelly extracellular matrix with the polyvinyl alcohol (PVA)-based hydrogel, demonstrated its ability in promoting chondrocyte adhesion and scaffold colonization [160]. Other studies are examining extracellular matrices developed from porcine articular cartilage [161]. Although these substrates worked as proper scaffolds for the growth of cells, their therapeutic and functional efficiency in cartilage regeneration still need to be proved. Other cell therapies include implanting chondrogenic lines differentiated from mesenchymal stem cells (MSC) into different polysaccharides or synthetic hydrogels. Collagen hydrogels have proved to provide an appropriate 3D environment for MSC chondrogenesis, isolated from Wharton’s jelly of human umbilical cord, and to be cytocompatible matrices with great potential for cartilage engineering [162].

One of the problems being reported when using cell therapies is the dedifferentiation of chondrocytes when cultured in two-dimensional cultures, making them less functional for cartilage repair. Wu L. et al. [163] hypothesized that functional exclusion of dedifferentiated chondrocytes can be achieved by detecting domains formation of collagen molecules deposited by chondrogenic cells into 3D environments. They reported a method which allows separation of functionally active chondrogenic cells, which produce high levels of collagen II, from functionally inferior dedifferentiated cells, which produce collagen X. To avoid dedifferentiation of cells once they are forming constructs, Lam J. et al. [164] investigated the ability of cell-laden bilayer hydrogels, by encapsulating chondrogenically and osteogenically pre-differentiated mesenchymal stem cells, by varying the period of chondrogenic pre-differentiation prior to implantation. Therefore, cell phenotype could be optimized in order to achieve ideal tissue repair. Furthermore, since regeneration of human articular cartilage is limited, various cellular sources have been studied, including adult and juvenile chondrocytes. Some studies have compared the formation of cartilage tissue, produced by juvenile, adult and osteoarthritic chondrocytes, inside 3D biomimetic hydrogels composed of poly(ethylene glycol) and chondroitin sulfate. It was found that after the cultured time, juvenile chondrocytes showed a greater upregulation of chondrogenic gene expression than adult chondrocytes, while OA chondrocytes showed a downregulation [101]. Another strategy being studied is the evaluation of therapeutic effects of intra-articular injections of hydrogels containing drugs used to treat osteoarthritis symptoms [165].

**Figure 5:**
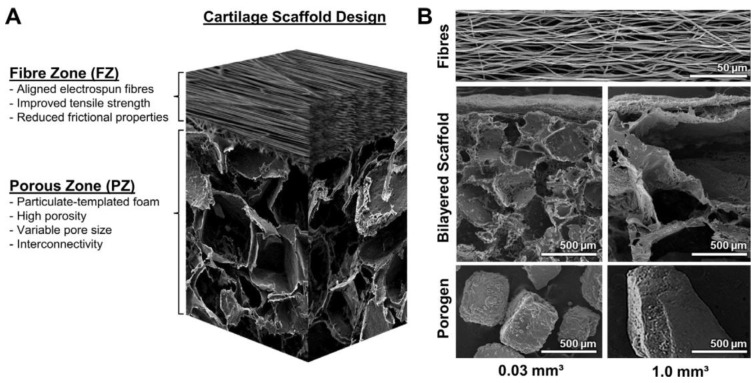
Bilayered cartilage scaffold (**A**) schematized by a diagram illustrating the electrospun fiber zone (FZ) deposited on a particulate-templated foam (PZ). The combination of the two distinct zones is designed to yield an anisotropic scaffold with a smooth articular surface and a more porous region for ECM deposition. (**B**) Electron microscopy images (top) of the aligned fiber zone that is shared between both scaffold varieties, (middle) the complete bilayered scaffolds with 0.3 mm^3^ (left) and 1.0 mm^3^ (right) pores, and (bottom) the sodium chloride porogens used to produce their respective scaffolds. Reprinted with permission from [169] J.A.M. Steele et al. Combinational scaffold morphologies for zonal articular cartilage engineering. *Acta Biomaterialia*. 10: 2065–2075.

Some other studies are meant to analyze how the structure and fabrication methods of the scaffolds influence cells behavior. Due to their intimate contact with chondrocytes, scaffolds are important components of cell niche. Investigations into micro-architecture of scaffolds have revealed that mean pore size is cell-type specific and influences cellular shape, differentiation and extracellular matrix secretion (Figure 5). Studies in collagen-hyaluronic acid scaffolds, fabricated with different mean pore size, showed that scaffolds with the largest mean pore size (300 µm) stimulated significantly a higher cell proliferation, chondrogenic gene expression and cartilage-like matrix deposition [166]. When using synthetic materials to produce hydrogels, it is necessary to determine their in vitro pore size and mechanical stiffness after being rehydrated, in order to predict their in vivo behavior. Hui J. H. et al. [167] found that freeze-dried oligo[poly(ethylene glycol)fumarate] (OPF) hydrogels with a pore size ranged from 20 to 433 µm in diameter and a mechanical stiffness of 1 MPa when rehydrated, enhance the formation of hyaline-fibrocartilaginous mixed tissue. However, these hydrogels, implanted alone into cartilage defects, are insufficient to generate a homogenously hyaline cartilage repair tissue. Kwon H. et al. [168] demonstrated that scaffolds, with different pore size and fabrication methods, influence the microenvironment of chondrocytes and their response to proinflammatory substances. Having high levels of proinflammatory cytokines can cause cartilage destruction and instability of the engineered cartilage tissue. These authors found that silk scaffolds with larger pore sizes support higher levels of cartilage matrix and leach more efficiently proinflammatory cytokines into the medium, influencing cartilage gene expression.

Furthermore, each zone of cartilage tissue varies in regard to biochemical content, morphology and biomechanical function. Deeper cartilage zones present higher stiffness, higher proteoglycan concentration but lower cellular density and same collagen concentration along cartilage tissue. In a general structural perspective, cartilage can be simplified into two main regions: (1) the superficial zone which exhibits a high tensile strength and low friction coefficient to keep a smooth articulation; (2) a dense ECM region rich in proteoglycan molecules which give the tissue adequate compressive mechanical properties by producing a high osmotic pressure within the tissue. Therefore, when fabricating a bilayer or three-layer scaffold, pore size and fabrication method of each layer influence the microenvironment of chondrocytes. As shown by Steele J.A.M. et al. [169], tissue engineering scaffolds can be designed to vary in morphology and function, offering a template: (1) to mimic the structural organization and functional interface of cartilage superficial zone; (2) to increase the extracellular matrix production; (3) to enhance the anisotropic mechanical properties. These authors fabricated a multi-zone cartilage scaffold by electrostatic deposition of polymer microfibers onto particulate-templated scaffolds with 0.03 mm^3^ and 1.0 mm^3^ porogens. They demonstrated that bilayered scaffolds can closely mimic some of the structural characteristics of native cartilage due to: (1) the addition of aligned fiber membranes enhances the mechanical and surface properties of scaffolds; (2) zonal analysis of scaffolds showed region-specific variations in chondrocyte number, sulfated GAG-rich extracellular matrix and chondrocytic gene expression; (3) smaller porogens (0.03 mm^3^) yield higher GAGs accumulation and aggrecan gene expression.

**Figure 6:**
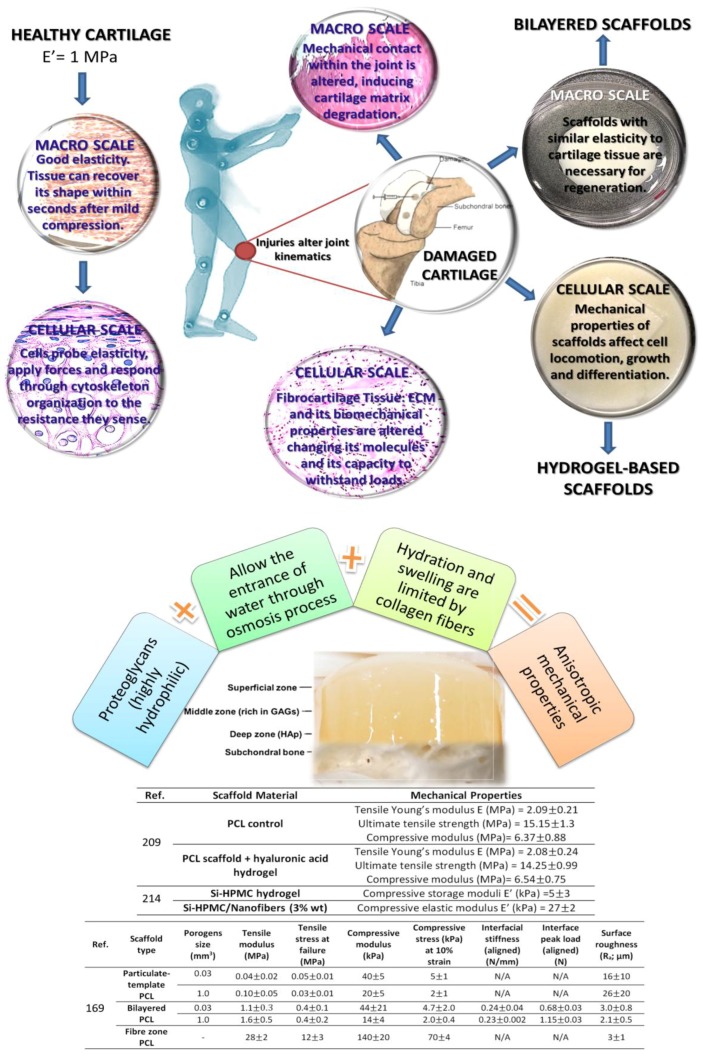
Correlation among the multi-scale biomechanical behavior of cartilage tissue. Original illustration designed and provided by the authors.

It is important to understand the multi-scale biomechanical behavior of cartilage tissue, in order to realize the connection among joint kinematics, tissue-level mechanics, cell mechanics and mechanotransduction, matrix mechanics and the nanoscale mechanics of matrix macromolecules. Therefore, understanding mechanical behavior at each scale helps to correlate cell biology, matrix biochemistry and tissue structure/function of cartilage (Figure 6).

Moreover, the combination of cell-based therapies with growth factor delivery, which can locally signal cells promoting their function, is also being investigated. Since morphogenetic protein (BMP-2) and transforming growth factor (TGF-β) are cytokines proposed as stimulants for cartilage repair, it is necessary to undertake a detailed comparative analysis of their biological effects on chondrocytes [170]. As an example, when chondrocytes are encapsulated in PEG hydrogels functionalized with transforming growth TGF-β1, proliferation and matrix production increase, in comparison with cells in hydrogels where TGF-β1 is dosed in the culture medium or untreated TGF-β1 hydrogels [171]. In another study, chondroitin sulfate-bone marrow adhesive hydrogel was used to localize and carry BMP-2 protein, which enhance articular tissue formation. It was demonstrated that these hydrogels were capable of supporting articular chondrocytes, viability and phenotype retention, stimulating cells to produce hyaline-like extracellular matrix [172]. Although expensive growth factors in cultures are used, there is still a production of cartilage with inferior mechanical and structural properties compared with the natural tissue. However, recent evidence suggests that GAGs incorporated into tissue engineering scaffolds can isolate and/or activate growth factors, mimicking better the natural extracellular matrix [173]. For example, in the presence of TGF-β3 releasing microspheres, gellan gum hydrogels facilitate a greater cell proliferation than fibrin or agarose hydrogels. Histological and biochemical analysis of these hydrogels indicated that fibrin hydrogel was the least chondro-inductive, while agarose and gellan gum hydrogels supported more robust cartilage formation because of a greater GAGs accumulation within the constructs. Unfortunately, gellan gum hydrogels stained more intensely for collagen type II and collagen type I, suggesting a fibrocartilaginous tissue phenotype [174]. There are other studies focused on calcified cartilage zone, which provides mechanical integration between articular cartilage and subchondral bone. Lee W.D. et al. [175] developed tissue-engineered osteochondral-like constructs with bone marrow stromal cells (BMSC), as single cell source. Cartilage tissue and a porous bone substitute substrate were formed with an interfacial zone of calcified cartilage. The authors found that the presence of calcified cartilage increased the shear load that the construct may withstand at the interface. However, preclinical studies are needed to determine if these osteochondral-like constructs could repair joint defects in vivo.

Evaluating cell-free and cell-based hydrogels reviewed above, only a few of these biomaterials have been used in clinical applications [45,46,74,176,177,178,179] because of four main unsolved problems in tissue engineering: (1) toxicity of some crosslinking agents [180]; (2) lack of mechanical integrity [156,181,182,183,184,185,186,187]; (3) poor control of gelation kinetics [188,189]; (4) unsuitable degradation kinetics [190,191,192,193,194].

Since some of the reactions used to synthesize hydrogels are limited due to their complexity, the use of cytotoxic reagents, instability of some functional groups, possible side reactions and low coupling efficiency, there is the need to explore and exploit simple and highly efficient methods which may be applicable to a great variety of biodegradable polymeric precursors.

## 3. Polysaccharides Versus Synthetic Hydrogels

The degradation rate and mechanical properties of manufactured hydrogels must be compatible with the growth of new tissue [109]. As mentioned before, hydrogels can be made of either natural or synthetic polymers, each of them with advantages and disadvantages, or even a combination of both, whether or not a reduction of disadvantages from the individual components can be obtained. When using natural precursors, good biocompatibility and bioactivity are ensured in the hydrogel scaffolds. However, there will be a high degradation rate, in contact with body fluids or medium, and a limited mechanical behavior, since natural polymer components are extracted from tissues and subsequently reconstructed to form hydrogels [173,195]. Nevertheless, the strength of these natural hydrogels can be increased by making the polymer matrix denser, using a chemical crosslinking or making chemical modifications. On the other hand, using synthetic precursors may provide appropriate physical and chemical properties for hydrogel scaffolds; however a good cell biological response and an adequate degradation rate may not occur [196,197]. The strength of the synthetic hydrogels can be raised by changing the molecular weight of the starting polymers, increasing polymer concentration or even the degree of functionalization, using reactive groups during the crosslinking reaction.

### 3.1. Manufacturing Techniques and Their Influence on Hydrogel Properties

Depending on the polymer precursors, hydrogels can be synthesized in different ways. In the first step a polymer is modified with a functional group; then the polymer is crosslinked, either physically or chemically, to form a three-dimensional structure. While chemical hydrogels are covalently crosslinked, physical hydrogels are not. Crosslinking can take place at the same time or after the copolymerization [198]. In situ crosslinked cytocompatible injectable hydrogels can be formed using: (1) non-toxic chemical crosslinkers, as in the Michael Addition Reaction, Click Chemistry, Schiff Base Reaction, and photo-crosslinking reactions; (2) enzymes for a biological crosslinking; (3) physical interactions, such as ionic and hydrophobic ones; (4) supramolecular chemistry utilizing self-assembly molecules [199].

The morphology and physico-chemical structure of hydrogels also depend on processing conditions applied during their formation, for example using electrospinning or cryogelation techniques. Hydrogel morphologies may range from fibrils, characteristic of protein-based hydrogels such as collagen and fibrin, to amorphous, characteristic of synthetic hydrogels such as PEG. When using the electrospinning technique, it is possible to obtain hydrogels with aligned fibrils morphologies. Mirahmadi F. et al. [200] added degummed chopped silk fibers and electrospun silk fibers to thermosensitive chitosan/glycerophosphate hydrogels, to reinforce scaffolds for hyaline cartilage regeneration. The results showed that mechanical properties of hydrogels were significantly enhanced; besides the composition of the scaffolds supported the chondrogenic phenotype. Nevertheless, when using cryogelation, because of ice crystals, a controlled porosity can be induced into hydrogels, helping them recovering their shape [201]. Another method to fabricate three-dimensional porous hybrid scaffolds for articular cartilage repair is combining freeze-dried [192,202,203] natural components with synthetic polymers, provide scaffolds with mechanical strength and an environment similar to natural ECM, to let chondrocytes proliferate. Lyophilization or freeze-drying technique produces highly porous structures with open pores throughout the scaffolds. The pores are introduced into the scaffolds, first by ice crystal formation, then by freeze-drying them. For this reason pore architecture reflects the ice crystal morphology [203]. Novel collagen/polylactide (PLA), chitosan/PLA, and collagen/chitosan/PLA hybrid scaffolds were fabricated by freeze-drying technique [204]. It was observed that collagen binds water inside the scaffold structure and it helps cells to penetrate into the hybrid scaffolds. To enhance anisotropic properties of cartilage scaffolds, aligned unidirectional pores can be formed depending on the real alignment of cells and the type of extracellular matrix that has to be repaired. Collagen-hybrid scaffolds, constructed by directional freezing, were studied. When varying freezing rates and suspension media, it is possible to obtain collagen-hybrid scaffolds with unidirectional pores, tunable pore sizes and pore morphologies [203]. The results demonstrated that directed horizontal ice dendrite formation and vertical ice crystal nucleation are responsible for aligned unidirectional ice crystal growth and, consequently, for aligned unidirectional pore structure of the collagen-hybrid scaffolds.

Since conventional fabrication techniques may not provide a precisely control of pore size, interconnectivity or pore geometry for scaffolds, solid freeform fabrication (SFF) techniques are now being used to produce 3D scaffolds with an organized interconnected pore structure which ensures good functionality and good mechanical strength, necessary to maintain new cartilage formation.

Bioprinting and plotting are being used as freeform fabrication techniques. These emerging techniques are used to fabricate viable 3D tissue constructs through a precise deposition of cells in hydrogels [205]. However, scaffolds, fabricated by these printing systems, often lack of flexibility and adequate mechanical properties [138,206]. Xu T. et al. [207] described the construction of a hybrid inkjet printing/electrospinning system that can be used to fabricate viable cartilage engineered tissue. They fabricated a five-layer construct, 1 mm thick, made of electrospun polycaprolactone fibers alternated with inkjet printing of rabbit elastic chondrocytes suspended in a fibrin-collagen hydrogel. One week after printing, evidence showed more than 80% of cell viability, cell proliferation and formation of cartilage-like tissue in the five-layer construct, both in vitro and in vivo assays, and demonstrated an improvement of mechanical properties, in comparison with printed alginate or fibrin-collagen hydrogels.

Novel techniques of tissue scaffold fabrication, as ultrafast pulse DLW lithography, are attractive due to their 3D structuring capability, spatial resolution, scaling flexibility and diversity of working materials.

### 3.2. Degradation Kinetics, Physical Properties (Applicability) and Biological Effects

Hydrogels degradation kinetics should be compatible with new tissue formation kinetics, in order to ensure a good integration of the construct. If hydrogels degrade very fast, it will trigger occurrence of defects in the formed tissue, such as cysts. On the other hand, if a very slow degradation occurs, hydrogels will inhibit the formation of new biological material and their integration with the surrounding tissue. Degradation of hydrogels can take place by either hydrolytic or enzymatic mechanisms. The hydrolytic degradation happens when hydrogel is kept in contact with fluids by breaking the polymer chains or the crosslinked network. This type of degradation mechanism can be controlled by limiting the amount of degradable precursor, used to synthesize hydrogels. The enzymatic degradation is caused by cells when they begin to invade the hydrogel or when encapsulated cells within the hydrogel start to proliferate or migrate throughout it. Many natural origin proteins have sites of cleavage in the protease, allowing hydrogels to degrade during the replacement of the ECM. This type of degradation may also depend on the degree and the type of chemical crosslinking in the hydrogels used as scaffolds [201].

### 3.3. Specificities of Polysaccharide-Based Hydrogels

When trying to manufacture biomimetic scaffolds for cartilage tissue regeneration, naturally-derived hydrogels are widely used, due to their macromolecular properties and because the employed biopolymers are part of the natural tissue that needs to be healed. Most of the studied naturally-derived hydrogels are based on biopolymers such as collagen, gelatin, chitosan, hyaluronic acid, chondroitin sulfate, agarose, alginate and fibrin [109,208].

### 3.4. Biological Response of Polysaccharide-Based Hydrogels

Since hyaluronic acid is a fundamental component of natural cartilage matrix, some studies have shown its importance and its good qualities as an excellent naturally derived polymer. Injections of hyaluronan into osteoarthritic joints have proved to restore viscoelasticity, augment joint fluid flow, normalize endogenous hyaluronan synthesis, and provide joint function [109,112]. Some other studies [198] have demonstrated that hyaluronic acid is favorable for cell response, by maintaining chondrogenic phenotype and increasing collagen type II production and angiogenesis during in vivo assays. Another example of an excellent natural polymer is chitosan. Chitosan can easily form polyelectrolyte complexes with hyaluronan and chondroitin sulfate [112,209]. Hu X. et al. [198] tried to mimic the natural cartilage extracellular matrix by synthesizing a biological hydrogel made of hyaluronic acid, chondroitin sulfate modified with 11-azido-3,6,9-tri-oxaundeca-1-amin and gelatin modified with propiolic acid, via click chemistry. Even though the molecular modifications made to the biopolymers let biological hydrogels have good response (making chondrocytes adhere and proliferate on them during in vitro assays), degradation process was too fast. They showed a loss of 45% w in 4 weeks, and a release of 20% w of gelatin and 10% w of chondroitin sulfate during the first two weeks, leading to macroscopic shrinkage of hydrogels.

Furthermore, when combining both naturally-derived and synthetically-derived polymers, adequate degradation kinetics and biological response can be achieved. Park H. et al. [129] created, by photocrosslinking, injectable hydrogels consisting of methacrylated glycol chitosan (MeGC) and hyaluronic acid. The photopolymerized hydrogels were cytocompatible. The incorporation of hyaluronic acid increased cell proliferation while encapsulated chondrocytes improved the cartilaginous extracellular matrix production. Following the same guideline, based on fabricating a hybrid scaffold containing both biological and synthetic components, B.R. Mintz et al. [209] studied a hybrid scaffold, made of a hyaluronic acid-based hydrogel combined with a porous poly(ε-caprolactone) material. They tried to understand better the interface and the potential for integration between tissue engineered cartilage scaffolds and surrounding native tissue. They noticed that precursors provide a microenvironment which supports chondrocyte infiltration and proliferation, while maintaining seeded phenotype and structural integrity over a 6-week culture period.

## 4. Future Trends: From Combination of Materials to Hybrid Hydrogels

**Figure 7:**
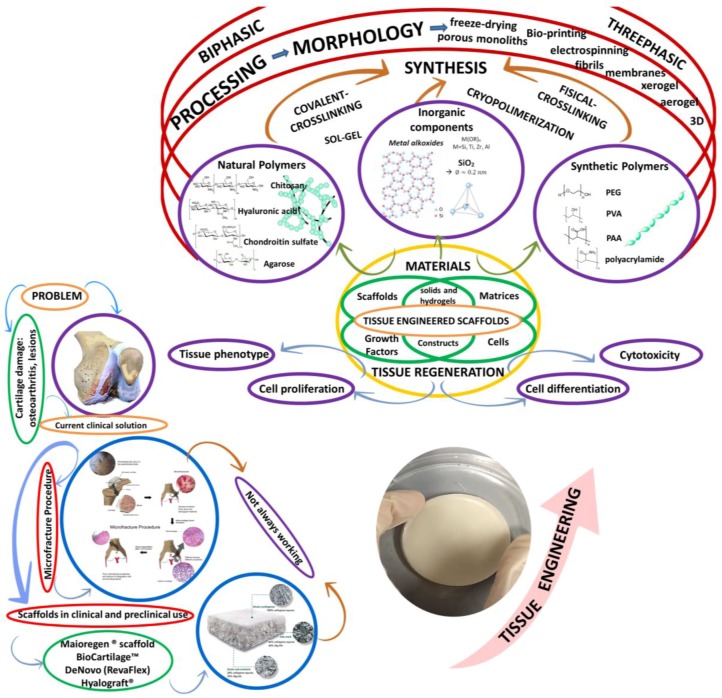
Correlation between current cell-based scaffolds used for cartilage regeneration. Original illustration designed and provided by the authors.

Identified problems hindering the application of hydrogels in cartilage regeneration, as described along this paper, include: mechanical properties [210] and mechanical instability [209,211]; dedifferentiation of chondrocytes [163]; toxicity of some of the used crosslinking agents [180]; poor control of gelation kinetics [188,189]; unsuitable degradation kinetics [190,191,192,193,194]. Figure 7 illustrates the combining requirements needed to create materials with biomimetic features.

Mechanical instability inhibits the integration of hydrogels with the surrounding native cartilage tissue when they are implanted [209,211]. In order to synthesize mechanically stable hydrogels and improve their mechanical properties, several options have been proposed. One of the most promising options relays on the principle that materials combination must show the ability to support matrix formation [210], as demonstrated by Boere K.W.M et al. [80]. In this research, it was determined that, when grafting two materials covalently (a 3-D-fabricated poly(hidroxymethylglycolide–*co*–ε–caprolactone)/poly(ε-caprolactone) thermoplastic polymer scaffold, functionalized with methacrylate groups and covalently linked to a chondrocyte-laden gelatin methacrylamide hydrogel), the binding strength between the materials improved significantly, resulting in the enhanced mechanical integrity of the reinforced hydrogel. Embedded chondrocytes in hydrogel scaffolds also showed significant cartilage-specific matrix deposition, both in vitro and in vivo assays.

Another promising option to enhance mechanical stability is by regenerating cartilage and bone tissue simultaneously using a two-phased scaffold, since ceramic-to-bone interface has a better and faster integration compared to hydrogel-to-cartilage interface [80]. Additionally it has been observed that bone integration is much faster than cartilage integration, occurring during 2 and 24 weeks after transplantation, respectively.

It is possible to have a stable fixation of a cartilage scaffold by exploring a fixation technique with the subchondral bone [206]. One way to accomplish this stable fixation is fabricating an osteochondral scaffold which facilitates fixation and integration with the surrounding cartilage tissue, accelerating the repair of defected articular cartilage when implanted. The general idea is that bone scaffolds act as anchors, providing mechanical stability for cartilage tissue regeneration, besides, the join between the bone component and the cartilage component should be strong enough to prevent dislocation or delamination on in vivo environment. In order to follow the theory mentioned above, Seol Y.-J. et al. [206] reinforced osteochondral scaffolds by developing combined scaffolds, made of hydrogel scaffolds anchoring to cartilage tissue and ceramic scaffolds anchoring to bone tissue. For in vivo assay, the combined scaffolds were press-fitted into osteochondral tissue defects, in rabbit knee joints. Hydrogel scaffolds and combined scaffolds were compared. After 12 weeks, in vivo experiments demonstrated that regeneration of osteochondral tissue, especially articular cartilage tissue regeneration, was better with combined scaffolds than with hydrogel scaffolds. Hydrogel scaffolds could not keep their initial position, suggesting that ceramic scaffolds in combined scaffolds provided mechanical stability for hydrogel scaffolds. Moreover, G. Camci-Unal et al. [202] realized that combined hydrogels can be biologically and physically tuned to yield within a range of different cell responses and, according to these responses, combined hydrogels may show potential therapeutic possibilities to treat either chondral or osteochondral lesions.

Following this trend, Yang S.S. et al. [138] developed a 3D plotting system to manufacture a biphasic graft which consists of cartilage and subchondral bone for application to osteochondral defects. A combined material (PLGH/alginate) was fabricated as supporting structure to induce a mature osteochondral graft. Cartilage-derived ECM or hydroxyapatite substances were blended with alginate and plotted together with human fetal cartilage-derived progenitor cells, either in the cartilage layer or in the subchondral bone one. The plotted biphasic osteochondral graft showed good integration between layers because no structural separation was observed, while there was dominant cartilage and bone tissue formation during differentiation assay. One of the limitations of using the osteochondral approach in combination with bone marrow derived MSCs is their terminal differentiation, as they seem to follow an endochondral ossification which can arrest differentiation at a stable cartilage hyaline-like phenotype during the chondrogenic process. For this reason, a chondrogenic stimulator, such as the recently described kartogenin which regulates Runx1 expression [212], has to be incorporated with a known inducer of chondrogenic differentiation and a suppressor of hypertrophy.

Another alternative to fulfill the inadequate mechanical strength of hydrogel is constructing a solid-supported thermogel, comprising hydrogel systems or demineralized bone matrix. Huang H. et al. [213] combined chitosan thermogel with demineralized bone matrix to produce solid-supported hydrogel scaffolds. This type of scaffolds provided sufficient strength for cartilage regeneration. They retained homogeneously more bone-derived mesenchymal stem cells (BMSCs) and they proved to have superior matrix production and chondrogenic differentiation in comparison with pure hydrogels and demineralized matrix by their own.

Using fibers of different natural materials to reinforce hydrogels is another way to offer mechanical strength to hybrid scaffolds [205,211]. Mechanical characteristics can be improved or modified using different strategies: (1) varying the number of fiber layers in the laminate; (2) combining different kinds of fibers and nanofiber sheets; (3) modifying crosslinking degree of hydrogels and fibers; (4) changing fibers content and surface treatment; (5) shifting fiber orientation. Fibers anisotropy is an excellent property to reach strong mechanical reinforcement at low charge levels. For example, Buchtová N. et al. [214] developed an injectable hydrogel based on a silanized cellulose derivative: hydroxypropyl methylcellulose interlinked with silica fibers. They proved that the compressive modulus of the hydrogel could be tunable, depending on the covalent bonding between biopolymer and silica fibers. In other approaches, the incorporation of bioactive species, such as cells, growth factors, peptides and proteins into the materials, is proposed to improve hydrogel scaffolds properties [73,215].

**Figure 8:**
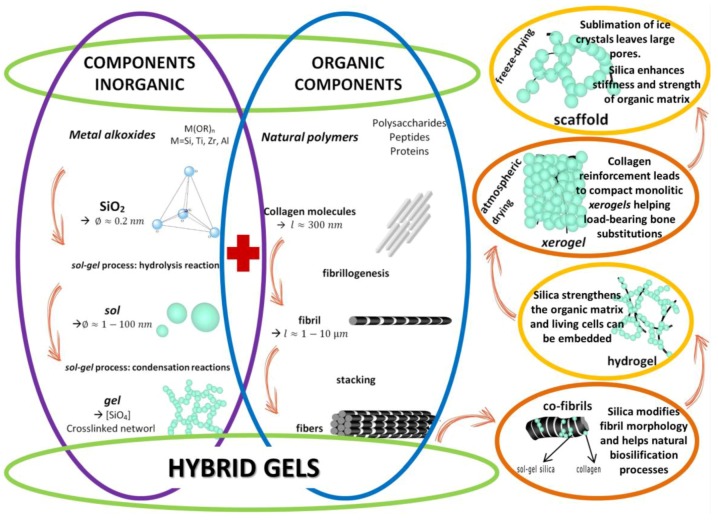
Advantages of hybrid *gels*. From soft mineralized hydrogels to hard compact *xerogels*. Original illustration designed and provided by the authors.

It can be deduced, from the reviewed studies, that combination of components yield up reinforced mechanical properties. However, to overcome the other requirements mentioned above, something different has to be done from the already investigated methods to fabricate materials. In authors’ opinion, there is a barely explored alternative in material science: studying the potential of hybrid hydrogels based on natural polymers and inorganic components. Hybrids are considered to be materials formed by two components bonded at a molecular level. Commonly one of these components is organic and the other one is inorganic. The new hybrid materials (Figure 8) may show superior characteristics in comparison with the two component phases. This possibility may offer a great potential to design new materials with the complex properties required for cartilage regeneration.

Since degradation rate and mechanical properties can be fine-tuned through chemical and/or physical modifications on either naturally or synthetically derived scaffolds, an excellent opportunity is given to hybrids to emerge as a promising solution for cartilage regeneration. When fine-tuned, hybrids can acquire amorphous, semicrystalline, hydrogen-bonded or supramolecular physical structures [109,210].

Hydrogels made from purely organic precursors are being used as viable materials for cartilage repair due to their ability to retain grate portions of water, swollen, distend and exhibit large changes of dimensions (volume changes of several- to 10-fold are common) [216,217]. These characteristics provide hydrogels with a low interfacial tension with water and other fluids, allowing them to reduce mechanical friction between tissues during implantation. The hydrophilic characteristics of hydrogels are caused by the presence of special hydrophilic molecules (–OH, –CONH, –CONH_2_, and –SO_3_H) found in the polymeric components. These molecules give hydrogels different absorption potential [216] and the ability to respond to a range of different stimuli, including temperature, pH, salt, specific (bio)chemical signals, and electric fields [217]. Nevertheless, hydrogels, based on a certain type of precursors, undergo uniform volumetric expansion and contraction, in response to several stimuli; therefore, the great added value of hybrid hydrogels is combining their precursors potential and restrictions. For example, to determine their degradation kinetics and enhanced biological and mechanical properties, it is possible to use: stiffer components like silica-based materials, which restrict swelling in hydrogels in certain positions or directions; or natural polysaccharide-based polymers hybridized with inorganic materials (hydroxyapatite, SiO_2_, or demineralized bone matrix), which restrict the number and type of hydrophilic molecules.

A further step may be the integration of nanotubes with different chemical composition into hybrid scaffolds which may provide them with bioactive and mechanical properties. This is the case of three-dimensional porous collagen sponges where single-walled carbon nanotubes are incorporated into [218]. The incorporation of single-walled carbon nanotubes improved cell proliferation and GAGs production in the in vivo microenvironment, because nanotubes were internalized by cells, with benefit for controlled and localized delivery of biological factors. Another study is the design of a biomimetic nanostructured composite cartilage scaffold, via biologically-inspired rosette nanotubes (RNTs) and biocompatible non-woven poly (l-lactic acid) (PLLA) [219]. It was concluded that, RNTs have a similar morphology with native collagen fibers when self-assembled in aqueous conditions, and besides they increase glycosaminoglycan, collagen and protein production; their nanotopography and surface chemistry enhance chondrogenic differentiation. Another study tested the biocompatibility of 3D artificial hexagonal-pore shaped hybrid organic-inorganic microstructured scaffolds in a rabbit model [220].

The association of silica and polysaccharides within composites or hybrids has demonstrated therapeutic benefits in a wide range of bio-inspired silica-collagen materials [221]. Although this kind of materials has been prepared over nearly 15 years, their application in cartilage regeneration treatments has not been exploited. The great value of hybrid materials (Figure 7) is that it can be synthesized a large variety of structures and properties, from soft mineralized hydrogels to hard compact xerogel, depending on the soft (cartilage) or hard (osteochondral) tissue wanted to be repaired or regenerated. Moreover, to fully comprehend these potential materials and to raise their value for the development of innovative biomedical devices for cartilage regeneration, it is important studying the interplay between the organic-inorganic precursors, which is to follow carefully the polymer self-assembly process and the inorganic condensation mechanisms. Therefore, biological, mechanical and degradation properties can be modulated, guaranteeing bioactivity, cytocompatibility, and an eventual biocompatibility.

## 5. Concluding Remarks

Currently, there are no clinical satisfactory solutions for cartilage tissue regeneration. From this problem, it raises the need for developing new types of materials and designing more suitable scaffolds which can be used for cartilage regeneration. Investigations based on cell-free hydrogel scaffolds have focused on the optimization of physical-chemical and mechanical properties of matrices. Whereas, cell-seeded hydrogel scaffold studies focus on whether or not they provide an appropriate biochemical and biomechanical environment for regenerating a long-lasting hyaline-type cartilage. Since many materials neither exhibit a low friction coefficient nor withstand several loading cycles, many combinations of polysaccharides and synthetic hydrogels have been assayed to obtain load-bearing and tribological properties similar to native cartilage tissue ones. However, some unsolved problems hinder the application of these materials to the clinic: (1) toxicity of some crosslinking agents; (2) lack of mechanical integrity; (3) poor control of gelation kinetics; (4) unsuitable degradation kinetics.

Among the most promising options, to synthesize mechanically stable hydrogels which support matrix formation, there is the combination of materials to regenerate cartilage and bone tissue simultaneously, using a two/several-phased scaffold. These combinations can be biologically and physically tuned to yield, within a range of different cell responses and according to these responses, combined hydrogels which may show potential therapeutic possibilities to treat either chondral or osteochondral lesions. Osteochondral treatment introduces a new problem and requires the use of a chondrogenic stimulator, such as the recently described kartogenin, to induce chondrogenic differentiation and suppress hypertrophy. From materials science perspective, hybrid hydrogels based on natural polymers and inorganic components may offer a fine tuning of mechanical and biological response, required to reproduce the complexity of the cartilage tissue environment. Silica, hydroxyapatite or demineralized bone matrix/polysaccharide-based hybrids restrict swelling in hydrogels in certain positions or directions by reducing the number and the type of hydrophilic molecules. The association of silica and polysaccharides within composites or hybrids has demonstrated therapeutic benefits in a wide range of bio-inspired silica-collagen materials. To fully comprehend these potential materials and to raise their value for the development of innovative biomedical devices for cartilage regeneration, it is important studying the interplay between the organic-inorganic precursors, which is to follow carefully the polymer self-assembly process and the inorganic condensation mechanisms. Therefore, biological, mechanical and degradation properties can be modulated, bioactivity and cytocompatibility guaranteed and biocompatibility eventually achieved.

## Figures and Tables

**Table 1:** Scaffolds in clinical and preclinical use for cartilage regeneration.

Application	Material	Problem-result	Ref.
Nose (dorsal augmentation material in rhinoplasty)	Tissue-engineered chondrocyte PCS (Porcine Cartilage-derived Substance) scaffold construct.	Preliminary animal study: Excellent biocompatibility, neocartilage formation starts. However, it was not confirmed that the constructs contributed to the formation of neocartilage.	[36]
Knee (subchondral bone)	Osteochondral biomimetic nanostructured scaffold Maioregen^®^	Better results in healing complex lesions in comparison with the implantation of a purely chondral scaffold.	[37]
	Cell-free biphasic scaffold: collagen-hydroxyapatite osteochondral scaffold	Statistically significant improvement in clinical scores. At 5 years, between 60.9% and 78.3% of the cases showed complete filling of the cartilage, complete integration of the graft, intact repaired tissue surface and homogeneous repaired tissue.	[38]
	Nanostructured biomimetic three-phasic collagen-hydroxiapatite construct	The implantations to treat chondral and osteochondral knee defects were effective in terms of clinical outcome, although MRI detected abnormal findings.	[39]
Knee (chondral defects)	Autologous ovine MNC Cell-seeded and cell-free dl-poly-lactide–*co*–glycolide (PLGA) scaffolds	The engineered tissue had not local or systemic adverse effects. However, only a poor integration of the tissue engineering product into adjacent tissue was reached and the formed ECM was not mature enough for long-lasting weight-loading resistance.	[40]
	Type I collagen-hydroxyapatite (Maioregen^®^) nanostructural biomimetic osteochondral scaffold	The use of the Maioregen^®^ scaffold is a good procedure for the treatment of large osteochondral defects; however, the lesion site seems to influence the results. Patient affected in the medial femoral condyle showed better results.	[41]
	DeNovo (RevaFlex) engineered tissue graft	Preliminary evidence suggests that DeNovo ET implant is capable of spontaneous matrix formation with no immune response, improving function and recreating hyaline-like cartilage.	[42]
Knee (femoral condyles)	Biphasic cylindrical osteochondral composite construct of dl-poly-lactide–*co*–glycolide (PLGA). Its lower body is impregnated with β-tricalcium phosphate (TCP)	The regenerated osteochondral tissue was evaluated as a tissue of acceptable quality. Regenerated cartilage was defined as being hyaline when the ground substance was homogeneous without fibrous texture.	[43]
Tibial plateau (osteochondral scaffold)	Osteochondral biomimetic collagen-hydroxiapatite scaffold (Maioregen^®^, Fin-ceramica, Faenza, Italy)	MRI abnormalities. Clinical outcome with stable results up to a mid-term follow-up.	[44]
Microfractured defect (for filling microfractures)	BioCartilageTM, product containing dehydrated, micronized allogeneic cartilage, implanted with the addition of platelet rich plasma	No human clinical outcomes data available. Data regarding results are limited to expert opinion.	[45]
	Chondroitin sulfate adhesive-Poly(ethylene glycol) diacrylate (PEGDA) hydrogel system combined with standard microfracture surgery	Significant increase in tissue fillers with defects in a short-term follow-up.	[46]
Knee (for donor site filling)	Artificial TruFit cylinders made of fully synthetic material called PolyGraft^®^-Material: 50% copolymer (PDLG), composed of 85% poly(d,l-lactide) and 15% glycolide; 40% calcium sulfphae, 10% PGA fibers	No clinical improvement could be found. The regeneration of the filled defects took more than 2 years, even though TruFit Plugs are supposed to stimulate cartilage and bone cell migration from the surrounding tissue to the synthetic cylinders.	[47]
	Porous poly(ethylene oxide)terephthalate/butylene terephthalate) (PEOT/PBT) implants	Treated defects did not cause postoperative bleeding. Well integration. Surface stiffness was minimally improved compared to controls. Considerable biodegradation after 9 months. Congruent fibrocartilaginous surface repair with interspersed fibrous tissue formation in implanted sites. Donor site: fibrocartilaginous surface repair.	[48]
Shoulder	Engineered hyaluronic acid membrane, Hyalograft^®^	Using the hyaluronic membrane had no effect on the final outcome. No difference was observed between the fibrocartilage tissue formed after implementing microfractures and the fibrocartilage tissue grown on the hyaluronic acid membrane scaffold.	[49]
